# Cold Electron Ionization (EI) Is Not a Supplementary Ion Source to Standard EI. It is a Highly Superior Replacement Ion Source

**DOI:** 10.1021/jasms.1c00241

**Published:** 2021-10-15

**Authors:** Aviv Amirav, Alexander B. Fialkov, Alexander Gordin, Oneg Elkabets, Ksenia J. Margolin Eren

**Affiliations:** † School of Chemistry, Tel Aviv University, Tel Aviv 6997801, Israel; ‡ Aviv Analytical Ltd., 24 Hanagar Street, Hod Hasharon 4527713, Israel

## Abstract

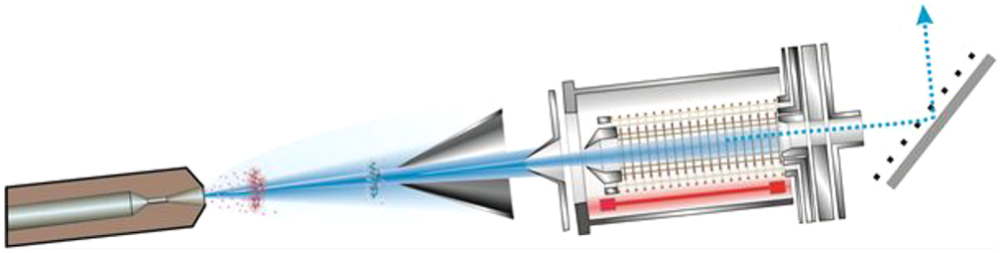

GC-MS usually employs a 70 eV electron ionization (EI) ion source, which provides mass spectra with detailed fragment ion information that are amenable for library search and identification with names and structures at the isomer level. However, conventional EI often suffers from low intensity or the absence of molecular ions, which reduces detection and identification capabilities in analyses. In an attempt to enhance the molecular ions, several softer ion sources are being used to supplement standard EI, including chemical ionization (CI), atmospheric pressure chemical ionization (APCI), field ionization (FI), photoionization (PI), and low electron energy EI. However, the most advantageous way to enhance molecular ions is to use cold EI, which employs 70 eV EI of cold molecules in supersonic molecular beams. Cold EI yields classical EI mass spectra with highly enhanced molecular ions, which still provides high detectability and library-searchable mass spectra. In this paper, we explain and discuss why cold EI is not a supplementary ion source to standard EI, but rather it is a highly superior replacement to standard EI. With cold EI, there is no need for standard EI or any other supplemental ion source. We describe 16 benefits and unique features of cold EI that not only yield better results for existing applications but also significantly extend the range of compounds and applications amenable for GC-MS analysis.

## Introduction

Sample detection and identification by GC-MS typically uses electron ionization (EI) with sample identification based on an EI mass spectra library database search. This approach excels in providing matching factors and identification probabilities of compounds with their names and structures including at the isomer level. However, such sample identification is also confronted by low intensity or the absence of molecular ions in many EI mass spectra, which impedes the effectiveness of library-based identification and prevents the possibility of getting elemental formula information. Thus, several softer ion sources are being used including chemical ionization (CI), atmospheric pressure chemical ionization (APCI), field ionization (FI), photoionization (PI), low electron energy EI, and EI of cold molecules in supersonic molecular beams (cold EI). Among these “soft” ion sources, CI is the most popular, and it is commercially available as an optional ion source from most GC-MS vendors.

However, we assert that EI of cold molecules in supersonic molecular beams (SMB), which we named cold EI, is far superior to all the known ionization approaches in GC-MS. Cold EI was initially developed in 1990,^1,2^ it is reviewed in refs (3 and 4), and a book on GC-MS with cold EI is being published.^5^ While CI and other “soft” ion sources serve as supplementary and/or complementary ion sources to standard EI, cold EI is uniquely advantageous to such an extent that with it there is no need for the standard EI ion source. If (rarely) desired, cold EI can also provide classical EI mass spectra via a mouse click mode of operation changing without any hardware change.

70 eV EI became the standard ionization energy for gas phase compounds in the early days of mass spectrometry as described in Fred Mclafferty’s classic book *Interpretation of Mass Spectra*.^6^ We note that the term “standard EI” is not well-defined since, while these ion sources use 70 eV electron energy, the resulting EI mass spectra can be somewhat different due to differences in the EI ion source structures and ion optics. As an example, the Agilent Inert and Extractor ion sources are different in their design from the Agilent high efficiency ion source (HES), and EI ion sources of TOF MS are different from those of quadrupole MS in both ion source structure and ion optics. However, they all provide about similar EI mass spectra that are compatible with library-based sample identification and therefore are defined as “standard EI”. Cold EI also provides library-searchable mass spectra, usually with enhanced molecular ions, which actually helps to increase the probability of accurate chemical identifications even if the matching factor may be slightly lower.

In this paper, we describe and explain why cold EI is far superior to standard EI.

## Experimental Section

To explore the various features of cold EI, including its effectiveness in enhancing molecular ions, we used the Aviv Analytical GC-MS with cold EI (model 5977-SMB, Aviv Analytical Ltd., Hod Hasharon, Israel). This instrument consists of an Agilent 5977 MSD (Agilent Technologies, Santa Clara, CA, USA) combined with the Aviv Analytical supersonic molecular beam (SMB) interface and its fly-through EI ion source for the electron ionization of internally cold molecules in the SMB (hence the name cold EI). We also developed the Tal-Aviv Molecule Identifier (TAMI) software that inverts the molecular ion isotope abundance patterns into elemental formulas.^7,8^ The standard EI and low eV EI experiments described below were performed with an Agilent 7890 GC and 5977 MS using 70 or 14 eV electron energies and other conditions as described in ref (9). GC-MS experiments with photoionization were performed with a Varian 1200 GC-MS (Varian Inc. Walnut Creek, CA, USA) that was modified to incorporate a photoionization ion source based on a deuterium discharge vacuum UV lamp, as described in ref (10).

## Results

**Figure 1:**
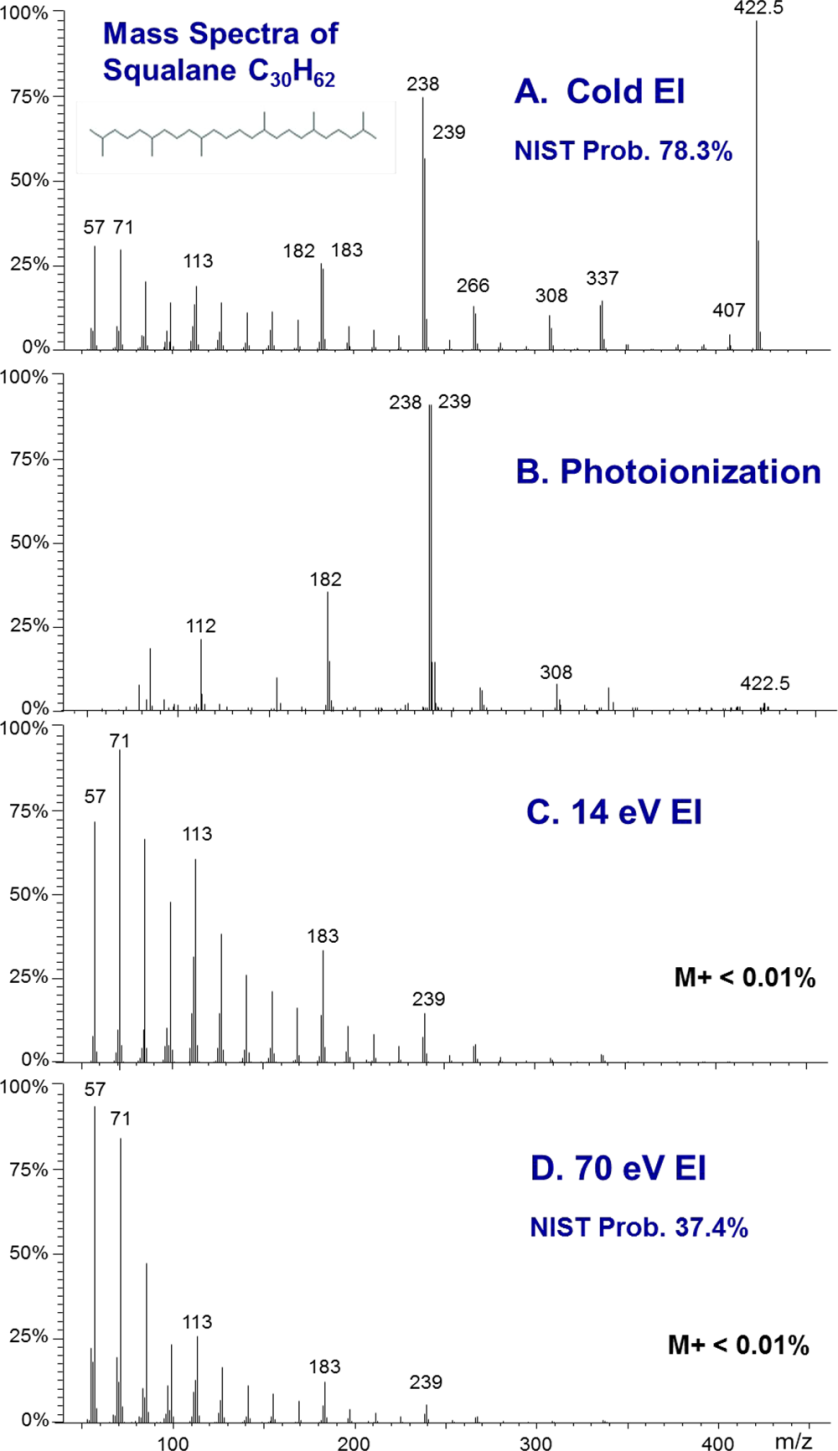
A comparison of mass spectra of the highly branched C_30_H_62_ squalane hydrocarbon, as obtained by cold EI (A, upper MS), photoionization (B, second MS), 14 eV EI (C, third MS), and 70 eV standard EI (D, bottom MS). The NIST library identification probabilities of cold EI and 70 eV standard EI mass spectra are also included.

In Figure 1, we show a comparison of mass spectra of the highly branched C_30_H_62_ squalane hydrocarbon as obtained by cold EI (upper MS), photoionization (second MS), 14 eV EI (third MS), and 70 eV standard EI (bottom MS). The NIST identification probabilities are also included for cold EI and 70 eV standard EI. As demonstrated, neither 70 eV standard EI nor “soft EI” at 14 eV exhibits any molecular ion as their molecular ions abundances are both weaker than 0.01%. Photoionization provides a weak molecular ion (∼2% relative abundance), whereas the molecular ion is the base MS peak in cold EI, and it also exhibits a clear isotopic pattern for M^+1^ and M^+2^. Furthermore, cold EI also provides a higher NIST identification probability of 78.3% compared with 37.4% of 70 eV standard EI, while photoionization failed to identify squalane by the NIST library. One reason why the PI mass spectrum failed to be identified by the NIST library is that it misses the low mass *m*/*z* = 57, 71, 85, 99 characteristic hydrocarbon fragmentation pattern. In addition, cold EI clearly (visibly) exhibits both the low mass fragment ions as well as highly amplified isomeric structural mass spectral information.

Accordingly, in cold EI, we can observe the *m*/*z* = 407 fragment ion, which indicates a side methyl loss and *m*/*z* = 337 that is formed via a loss of C_6_H_13_ radical which hints to the position of another side methyl group, and similarly all other high mass fragment ions are structurally informative. We note that the 14 eV low eV mass spectrum is very similar to the 70 eV standard EI mass spectrum, and only minor differences in the relative intensities of the low mass fragment ions are exhibited. The reason for this observation is that the molecular ion fragmentation is dominated by the large internal thermal energy of C_30_H_62_. Thus, despite some claims to the contrary, low eV EI (e.g., 14 eV) is not a “soft” ionization approach, especially in contrast to cold EI which yields a molecular ion abundance 10 000 times greater while still providing full mass spectral fragment ions information and compatibility with standard EI library-based identification. Figure 1 is not unusual, and it demonstrates how cold EI is far superior to standard EI, low eV EI, and PI in the mass spectral information provided. Reference (9) exhibits and describes 46 other examples of compounds from several different compound classes analyzed by cold EI in comparison with both 70 eV standard EI and low eV EI.

## Discussion

Among the “soft” ion sources, CI is by far the most common, largely because it is sold by all the major GC-MS providers. Unlike cold EI, the use of CI: (1) requires time-consuming venting and physical ion source replacement; (2) adds to the overall instrument cost, including the need for a CI gas such as methane and its related safety issues; (3) yields a signal that is typically 100-fold lower than of standard EI; (4) it is ineffective with some compounds such as hydrocarbons; (5) provides mass spectra that are incompatible with EI-MS library-based identification; (6) its range of compounds amenable for analysis is reduced due to extended CI ion source peak tailing and degradation; (7) often provides distorted molecular ion isotope abundances, which reduces the possibility of identification based on obtaining an elemental formula with unit mass resolution quadrupole MS instruments. Thus, despite the clear need for having trustworthy molecular ions, CI is not widely (rarely) used.

GC-MS ion sources are characterized by several features, most of which are rarely discussed or compared. Sixteen important features are described in the list below, including important yet sometimes unexpected benefits of enhanced molecular ions in cold EI that make it the best and most effective GC and MS interface and ion source:

(1) **Molecular ion enhancement**. Cold EI provides enhanced molecular ions for the broadest range of compounds.^3−5,9^ The degree of molecular ion enhancement can be minimal for small molecules, while it can be unexpectedly large for large molecules, often exceeding enhancement factors of 1000 for hydrocarbons with the carbon number above C_36_H_74_ or for branched isomers greater than C_30_H_62_ as shown in Figure 1 for squalane. This enhancement factor of cold EI is much higher than of 14 eV “low eV” EI or of photoionization.
(2) **Particular importance for large compounds**. The molecular ion abundances strongly decline with mass in standard EI, and for hydrocarbons they decline by 20% for each added carbon atom, while in cold EI the high molecular ion abundance is about size independent.^5^ The reason for this observation in standard EI is that the intramolecular vibrational energy heat capacity increases about linearly with the number of atoms in the sample compound. Thus, while the molecular ion enhancement in cold EI for small molecules may be minimal, it can exceed a factor of 1000 for C_36_H_74_ or bigger hydrocarbons. In contrast, CI and/or APCI are ineffective for the analysis of hydrocarbons, and as already described^5,9^ low eV EI does not enhance the molecular ions of large hydrocarbons.
(3) **The most trustworthy molecular ions**. Cold EI eliminates vacuum background and has no adduct ions, and it reduces column bleed and ghost peaks via enabling lower elution temperatures at high column flow rates. Thus, cold EI makes it much easier to visually identify the molecular ions compared to standard EI, low eV EI, PI, CI, and/or APCI. Accordingly, cold EI provides the highest ratio of molecular ions to higher mass noise ions, and thus the cold EI molecular ions are the most trustworthy.^4,5^
(4) **Compatibility with NIST library identification**. Cold EI provides both enhanced molecular ion and library-searchable fragment ions, and thus *it is the only “soft” ionization method that is compatible with NIST library identification*. While the molecular ion is enhanced, cold EI also results in the formation of fragment ions that are formed from doubly charged molecular ion dissociation into nonstatistical fragment ions. These fragment ions are not present at low electron energies (low eV EI).
(5) **Improved NIST library identification**. The molecular ion provides the most characteristic information about the analyzed sample compound, and by enhancing the intensity of the molecular ion, cold EI actually improves the NIST library identification probabilities. Knowledge of the sample compound mass also leads to a better ability to reject incorrect identifications. This aspect, including computer simulations of the improvement of library identification probabilities with enhanced molecular ions, has been described previously in detail.^9,11^
(6) **NIST library identification confirmation via availability of molecular ions**. Trustworthy molecular ions provide an independent confirmation to the NIST library identification regardless of its provided matching factors and identification probabilities. Without having trustworthy molecular ions, the library identification often fails and/or cannot be trusted. Accordingly, with cold EI, we always look at the molecular ion to see if it is the same as in the NIST library #1 compound in its list.
(7) **Provision of elemental formula**. The cold EI enhancement of the molecular ions combined with exhibiting accurate isotope abundances enables the effective use of the Tal-Aviv Molecule Identifier (TAMI) software with unit resolution quadrupole MS data for the conversion of isotope abundances into the elemental formula.^7,8^ We note that in CI and/or APCI, the isotope abundances could be distorted due to incomplete proton transfer. In addition, cold EI enables the analysis of extended range of compounds from which greater portions are not in the library and thus require having the elemental formula as the best form of tentative identification.
(8) **Extended range of compounds amenable for analysis**. Cold EI enables a significantly greater range of compounds amenable for analysis, including low volatility, polar, and those that require derivatization.^5,12^ This feature is achieved via the possible use of short columns with higher carrier gas flow rates (up to 100 mL/min) that result in lower elution temperatures from the injector to the column and from the column to the SMB, while ion source-related degradation is fully eliminated in cold EI via its fly-through ion source.^3−5,12^ CI for example has a closed ion source configuration (compared with standard EI) to increase the internal CI gas pressure, and thus it exhibits an early onset of ion source-related peak tailing and as a result it is prone to a smaller range of compounds amenable for analysis. *We view this extended range benefit of cold EI as its most important feature* as it results in largely extended range of cold EI applications that bridges the gap between GC-MS and LC-MS.
(9) **One ion source for all types of analyses**. Cold EI can serve for the full range of all GC-MS with standard EI applications, including additional applications that are unique to cold EI. Thus, with cold EI there is no need to have any other ion source, while in contrast most other “soft” ion sources are supplementary and optional to standard EI.^3−5^
(10) **Fast Cold EI-Classical EI ion sources mode changing**. In the rare case of a desire to have classical EI mass spectra, the cold EI ion source enables its replacement to “standard EI” like an ion source with a simple method change that results in the reduction of the cooling helium makeup gas flow rate (combined with column flow rate) from 60 mL/min to 5 mL/min. This change can be achieved even in the middle of an analysis run.^4^ CI, PI, or FI typically require venting and manual ion source replacement which adds to the cost; plus, CI requires additional CI gas and its safety requirements, while APCI cannot be replaced with standard EI. Low eV EI is ineffective for compounds which do not exhibit molecular ions.
(11) **Cold EI improves MS-MS**. The molecular ions are the most selective ions against matrix interference which is reduced with mass by about a factor of 10 per 100 u.^13^ Furthermore, the product ions that are produced from the molecular ion CID also typically have a higher mass, and thus MS-MS on the molecular ion can be 100 times more selective than MS-MS on a fragment ion. Accordingly, GC-MS-MS with cold EI is much more sensitive in complex matrix analysis than with standard EI, in analogy to MS-MS with electrospray LC-MS. While this improved selectivity is also true for other “soft” ion sources, cold EI provides greater instrumental sensitivity with MS-MS than any other “soft” ion source.^14^ This feature and the ability to extend the range of compounds amenable for GC-MS make cold EI ideal for GC-MS-MS.
(12) **Ion source-related peak tailing and degradation**. Cold EI involves the ionization of cold molecules during their flight path through a contact-free fly-through ion source. Thus, any ion source-related degradation or peak tailing is fully and inherently eliminated. On the other hand, peak tailing and ion source degradation adversely affect standard EI, low eV EI, CI, and PI. Thus, compounds with OH, NH, or COOH often require derivatization for their analysis by GC-MS and otherwise may exhibit a nonlinear response and high limit of detection.^15^ In CI, the closed ion source causes both peak tailing and degradation to be further promoted and amplified.
(13) **Response uniformity**. Cold EI is unique in exhibiting an approximately uniform compound independent response^3−5,16^ that enables semi-quantification without external calibration. Standard EI exhibits a uniform response to small molecules, but it loses this feature for large compounds due to ion source-related peak tailing. Analytes with OH, COOH, or NH groups also need external calibration due to ion source-related peak tailing and degradation that also result in nonlinear response. Because of its closed ion source structure, CI is worse than standard EI in this respect, while low eV EI is worse than 70 eV standard EI due to its electron energy being close to the compound-dependent ionization potentials.
(14) **Superior sensitivity**. The cold EI molecular ions in large compounds provide superior sensitivity in reconstructed single ion monitoring (RSIM) than standard EI in view of both a higher signal and lower vacuum background and column bleed noise.^4,5^ The lower column bleed is due to lower elution temperatures in cold EI analysis at higher column flow rates.^12^ The cold EI signal (TIC and molecular ions) is far greater than that of CI, FI, PI, or low eV EI. CI for example provides a 10–1000 times weaker signal than 70 eV standard EI, depending on the analyte proton affinity. For example, S/N specifications of the Agilent 5977 MSD call for EI to be 125-fold more sensitive than CI. While cold EI S/N can be equivalent to that of 70 eV standard EI with OFN (cold EI S/N for 1 pg OFN at *m*/*z* = 272 is >10^+6^ due to zero noise and its LOD in SIM is 0.2–0.4 fg), it is superior with cold EI for large compounds, and the greater the difficulty in standard EI analysis, the greater is the gain in cold EI S/N. For example, cold EI can provide a factor of 1000 greater S/N in single ion monitoring of the molecular ions for cholesterol and *n*-C_32_H_66_ in comparison with the Agilent high efficiency ion source (HES).^4,5^ Furthermore, many compounds can be effectively ionized by cold EI, while they are not amenable for analysis by standard EI.^12^
(15) **Improved isomer identification**. The combination of enhanced molecular ions and high mass fragment ions provides extended structural and isomer information, as demonstrated in Figure 1 for squalane and in a few other examples for improved structural mass spectral information in ref (9). Thus, cold EI is the best ion source for isomer characterization.
(16) **Isomer distribution analysis**. Enhanced molecular ions enable the unique method of isomer distribution analysis for fuels and oils characterization and optimization.^17^ This is a potentially very important application that requires having abundant molecular ions for branched hydrocarbon isomers, which is not shared by standard EI, low eV EI, and PI, as demonstrated in Figure 1.

Consequently, GC-MS with cold EI is superior in sample identification and quantification versus GC-MS with any other type of ionization method and ion source. Thus, cold EI does not serve to merely complement standard EI, but rather, it should be used to replace standard EI as a far superior ion source and GC-MS interface.

We view the cold EI feature of extended range of compounds and applications amenable for analysis as its most important feature that bridges the gap between GC-MS and LC-MS, while having a trustworthy enhanced molecular ion is also a highly important benefit of cold EI. Thus, cold EI can lead the way to the future of GC-MS, and with it there is no need for any additional ion source, while all the central GC-MS performance features are improved.

## References

[ref1] Amirav A.; Danon A. Electron Impact Mass Spectrometry in Supersonic Molecular Beams. Int. J. Mass Spectrom. Ion Processes 1990, 97, 107–113. 10.1016/0168-1176(90)85042-Z.

[ref2] Amirav A. Electron Impact Mass Spectrometry of Cholesterol in Supersonic Molecular Beams. J. Phys. Chem. 1990, 94, 5200–5202. 10.1021/j100376a002.

[ref3] Amirav A.; Gordin A.; Poliak A.; Fialkov A. B. Gas Chromatography Mass Spectrometry with Supersonic Molecular Beams. J. Mass Spectrom. 2008, 43, 141–163. 10.1002/jms.1380. 18225851

[ref4] Amirav A.; Fialkov A. B.; Margolin Eren K. J.; Neumark B.; Elkabets O.; Tsizin S.; Gordin A.; Alon T. Gas Chromatography–Mass Spectrometry (GC–MS) with Cold Electron Ionization (EI): Bridging the Gap Between GC–MS and LC–MS. Current Trends in Mass Spectrometry, Supplement to LCGC North America 2020, 18, 5–15.

[ref5] Amirav A. Gas Chromatography - Mass Spectrometry with Cold EI: Leading the Way to the Future of GC-MS; Scientific Research Publishing Inc., USA, 2021. ISBN: 978-1-64997-142-5.

[ref6] Mclafferty F. W. Interpretation of Mass Spectra; W. A. Benjamin, Inc., 1966.

[ref7] Alon T.; Amirav A. Isotope Abundance Analysis Method and Software for Improved Sample Identification with the Supersonic GC-MS. Rapid Commun. Mass Spectrom. 2006, 20, 2579–2588. 10.1002/rcm.2637. 16897787

[ref8] Alon T.; Amirav A. A Comparison of Isotope Abundance Analysis and Accurate Mass Analysis in their Ability to Provide Elemental Formula Information. J. Am. Soc. Mass Spectrom. 2021, 32, 929–935. 10.1021/jasms.0c00419. 33779170 PMC8154599

[ref9] Margolin Eren K. J.; Elkabets O.; Amirav A. A Comparison of Electron Ionization Mass Spectra Obtained at 70 eV, Low Electron Energies and with Cold EI and Their NIST Library Identification Probabilities. J. Mass Spectrom. 2020, 55, e4646 10.1002/jms.4646. 32996658

[ref10] Fialkov A. B.; Ikonen E.; Laaksonen T.; Amirav A. GC-MS with Photoionization of Cold Molecules in Supersonic Molecular Beams – Approaching the Softest Ionization Method. J. Mass Spectrom. 2020, 55, e4516 10.1002/jms.4516. 32567120

[ref11] Alon T.; Amirav A. How Enhanced Molecular Ions in Cold EI Improve Compound Identification by the NIST Library. Rapid Commun. Mass Spectrom. 2015, 29, 2287–2292. 10.1002/rcm.7392. 26522322

[ref12] Fialkov A. B.; Gordin A.; Amirav A. Extending the Range of Compounds Amenable for Gas Chromatography Mass Spectrometry Analysis. J. Chromatogr. A 2003, 991, 217–240. 10.1016/S0021-9673(03)00247-4. 12741601

[ref13] Kochman M.; Gordin A.; Goldshlag P.; Lehotay S. J.; Amirav A. Fast, High Sensitivity, Multi-Pesticide Analysis of Complex Mixtures with the Supersonic GC-MS. J. Chromatogr. A 2002, 974, 185–212. 10.1016/S0021-9673(02)01245-1. 12458937

[ref14] Hejazi L.; Ebrahimi D.; Hibbert D. B.; Guilhaus M. Compatibility of electron ionization and soft ionization methods in gas chromatography/orthogonal time-of-flight mass spectrometry. Rapid Commun. Mass Spectrom. 2009, 23, 2181–2189. 10.1002/rcm.4131. 19530152

[ref15] Amirav A.; Keshet U.; Belgorodsky B. Linearity Sensitivity and Response Uniformity Comparison of the Aviv Analytical 5975-SMB GC-MS with Cold EI and the Agilent 5977A GC-MS with Standard EI. Advanced GC-MS Blog Journal, May 8, 2014. http://blog.avivanalytical.com/2014/05/linearity-sensitivity-and-response.html.

[ref16] Amirav A.; Gordin A.; Hagooly Y.; Rozen S.; Belgorodsky B.; Seemann B.; Marom H.; Gozin M.; Fialkov A. B. Measurement and Optimization of Organic Chemical Reaction Yields by GC-MS with Supersonic Molecular Beams. Tetrahedron 2012, 68, 5793–5799. 10.1016/j.tet.2012.05.031.

[ref17] Fialkov A. B.; Gordin A.; Amirav A. Hydrocarbons and Fuels Analysis with the Supersonic GC-MS – The Novel Concept of Isomer Abundance Analysis. J. Chromatogr. A 2008, 1195, 127–135. 10.1016/j.chroma.2008.04.074. 18495139

